# Correlation of optic nerve sheath diameter with directly measured intracranial pressure in Korean adults using bedside ultrasonography

**DOI:** 10.1371/journal.pone.0183170

**Published:** 2017-09-13

**Authors:** Jin Pyeong Jeon, Si Un Lee, Sung-Eun Kim, Suk Hyung Kang, Jin Seo Yang, Hyuk Jai Choi, Yong Jun Cho, Seung Pil Ban, Hyoung Soo Byoun, Young Soo Kim

**Affiliations:** 1 Department of Neurosurgery, Hallym University College of Medicine, Chuncheon, Korea; 2 New Frontier Research Institute, Hallym University College of Medicine, Chuncheon, Korea; 3 Department of Neurosurgery, Seoul National University Bundang Hospital, Seongnam, Korea; 4 Department of Emergency Medicine, Seoul Emergency Operations Center, Seoul, Korea; 5 Department of Anesthesiology and Pain Medicine, Veterans Health Service Medical Center, Seoul, Korea; University of Houston, UNITED STATES

## Abstract

**Objectives:**

The correlation of optic nerve sheath diameter (ONSD) as seen on ultrasonography (US) and directly measured intracranial pressure (ICP) has been well described. Nevertheless, differences in ethnicity and type of ICP monitor used are obstacles to the interpretation. Therefore, we investigated the direct correlation between ONSD and ventricular ICP and defined an optimal cut-off point for identifying increased ICP (IICP) in Korean adults with brain lesions.

**Methods:**

This prospective study included patients who required an external ventricular drainage (EVD) catheter for ICP control. IICP was defined as an opening pressure over 20 mmHg. ONSD was measured using a 13 MHz US probe before the procedure. Linear regression analysis and receiver operator characteristic (ROC) curve were used to assess the association between ONSD and ICP. Optimal cut-off value for identifying IICP was defined.

**Results:**

A total of 62 patients who underwent ONSD measurement with simultaneous EVD catheter placement were enrolled in this study. Thirty-two patients (51.6%) were found to have IICP. ONSD in patients with IICP (5.80 ± 0.45 mm) was significantly higher than in those without IICP (5.30 ± 0.61 mm) (*P* < 0.01). The IICP group showed more significant linear correlation with ONSD (r = 0.57, *P* < 0.01) compared to the non-IICP group (r = 0.42, *P* = 0.02). ONSD > 5.6 mm disclosed a sensitivity of 93.75% and a specificity of 86.67% for identifying IICP.

**Conclusion:**

ONSD as seen on bedside US correlated well with directly measured ICP in Korean adults with brain lesions. The optimal cut-off point of ONSD for detecting IICP was 5.6 mm.

## Introduction

Urgent diagnosis of acute increased intracranial pressure (IICP) and prompt treatment are required to avoid poor clinical outcomes. Invasive surgical procedures such as ICP monitor probe insertion in the brain parenchyma or ventricles are standard modalities for measuring ICP. However, procedure-related complications such as infection, hemorrhage or catheter malfunction occur at a rate between 6% and 32.8% [1, 2]. In addition, the procedures are not widely known by many doctors who work at emergent departments (ER) or intensive care units (ICU), except for neurosurgeons.

The feasibility of using optic nerve sheath diameter (ONSD) as seen on ultrasonography (US) as a non-invasive method to identify IICP is increasingly reported [3–5]. Several studies have reported a correlation between ONSD and ICP, and they obtained normal ONSD with a mean range of 3.5–5.1 mm and a cut-off value in the range of 4.1–5.7 mm for identifying IICP. Nevertheless, differences in ethnicity and type of ICP monitor used are obstacles to interpretation and usage of the correlation in an East Asian population. Regarding ethnic differences [6], most previous results were obtained from Western population, not East Asians. Therefore, these results may not accurately reflect the relationship between IICP and ONSD in an East Asian population. Although recent studies were conducted on Korean and Chinese populations, they did not show a correlation between ONSD and directly measured ICP [6–9]. Regarding the type of ICP monitor, though intraventricular ICP monitors such as EVD have been suggested to be the gold standard to measure global ICP, most previous articles used parenchymal ICP monitoring, which tends to reflect focal ICP, or a combination of parenchymal ICP monitoring with intraventricular monitoring [2, 10]. Additionally, when there is a large hematoma which requires prompt decompression surgery, an ICP monitoring probe can be placed after hematoma removal, which leads to underestimation of ICP as compared with ICP at the time of initial presentation. For these reasons, existing studies may not have accurately correlated global ICP with ONSD. Accordingly, we carried out this study in Korean adult patients who required measurement of ICP to determine the relationship between simultaneous US measurements of ONSD and directly measured ICP using ventricular ICP monitoring.

## Methods

### Study setting and population

This prospective study was performed from April 2015 to October 2016 in two institutions. The inclusion criteria were as follows: 1) age > 18 years, 2) abnormal results on brain computed tomography (CT) or magnetic resonance imaging (MRI), 3) patients who required EVD, and 4) patients who were admitted to the ED or ICU. The exclusion criteria were as follows: 1) age < 18, 2) significant ocular trauma, 3) mass lesions in the orbital area or cavernous sinus, such as an arachnoid cyst, and 4) severe mass effect due to a large hematoma or tumor requiring decompression surgery before placement of an ICP monitoring probe.

### Measurement of ONSD and ICP

Ocular US was conducted in standard ophthalmic B-mode using 13 MHz US probe (ProSound Alpha 6, Hitachi Medical Corp., Tokyo, Japan) on closed eyelids. The probe was applied on the temporal part of the closed upper eyelid with coupling gel [11] [12]. For each patient, investigator performed two measurements on each eye in transverse plane. The resulting four measurements were then averaged to yield a mean ONSD to minimize intraobserver variability. The procedure was performed by two investigator two investigators (JP-J and SU-L). EVD was performed under general anesthesia with remifentanyl and sevoflurane, a volatile anesthetic with fast onset and no significant influence on ICP [13, 14]. Initial US ONSD was measured under general anesthesia before placement of the EVD catheter (10.5 Fr EVD catheter, Yushin Medical Co., Korea). The opening pressure, determined just after placement of the EVD catheter, was used as the initial ICP, and IICP was defined as an ICP over 20 mmHg. Written informed consent was obtained from each patient or legal guardian. This study was approved by the Institutional Review Boards (IRB) of participating institutions (Hallym University College of Medicine, IRB No., 2015–131; Seoul National University Bundang Hospital, IRB No, H-1309-004-515).

### Statistical analysis

Categorical variables are presented as numbers and percentages. Continuous data are shown as the mean ± standard deviation (SD). Intra-observer and inter-observer variability was used using intra-class correlation coefficient (ICC) analysis. Scatter plot and linear regression analysis were used to assess the relationship between ONSD and ICP. Student’s t-test was carried out to compare mean ONSD values according to the presence of IICP. Furthermore, the cut-off point, sensitivity, and specificity of US ONSD measurement to identify IICP were calculated. A receiver operator characteristic (ROC) curve was generated to determine the cut-off point that optimized sensitivity and specificity. A *P*-value < 0.05 was regarded as statistically significant. Statistics were performed using SPSS version 22 (SPSS, Chicago, IL) and MedCalc (www.medcalc.org).

## Results

A total of 62 ONSD measurements were performed for 62 patients in this study. Thirty-two individuals (51.6%) had IICP as defined as an ICP value > 20 mmHg. Detailed information on the clinical characteristics of the patients is given in Table 1. Twenty patients were male and the mean age of all patients was 55.8 ± 14.3 years (21 to 83 years). Of the 62 patients, 38 (61.3%) had intracerebral hemorrhaging (ICH) and 13 (21.0%) were diagnosed with subarachnoid hemorrhaging. A brain tumor or abscess was found in 5 patients (8.1%), and 4 patients (6.5%) had intraventricular hemorrhaging due to arteriovenous malformation (AVM) or moyamoya disease (MMD).

**Table 1:** Baseline characteristics of patients used in this study (n = 62).

Variables	IICP (n = 32)	Without IICP (n = 30)
Age (years)	54.4 ± 15.8	57.4 ± 12.6
Male (%)	8 (25.0%)	12 (40.0%)
HTN (%)	17 (53.1%)	14 (46.7%)
DM (%)	3 (9.4%)	6 (20.0%)
Dyslipidemia (%)	9 (28.1%)	10 (33.3%)
Presentation		
Altered mentality	23 (71.9%)	20 (66.7%)
Headache	5 (15.6%)	7 (23.3%)
Motor weakness	4 (15.5%)	3 (10.0%)
Diagnosis		
Intracerebral hemorrhage	20 (62.5%)	18 (60.0%)
Subarachnoid hemorrhage	6 (18.8%)	7 (23.3%)
Tumor / Abscess	4 (12.5%)	1 (3.3%)
Intraventricular hemorrhage	2 (6.3%)	2 (6.7%)
Hydrocephalus	0 (0%)	2 (6.7%)

Continuous data is presented as mean ± SD. IICP, increased intracranial pressure; HTN, hypertension; DM, diabetes mellitus

**Fig 1:**
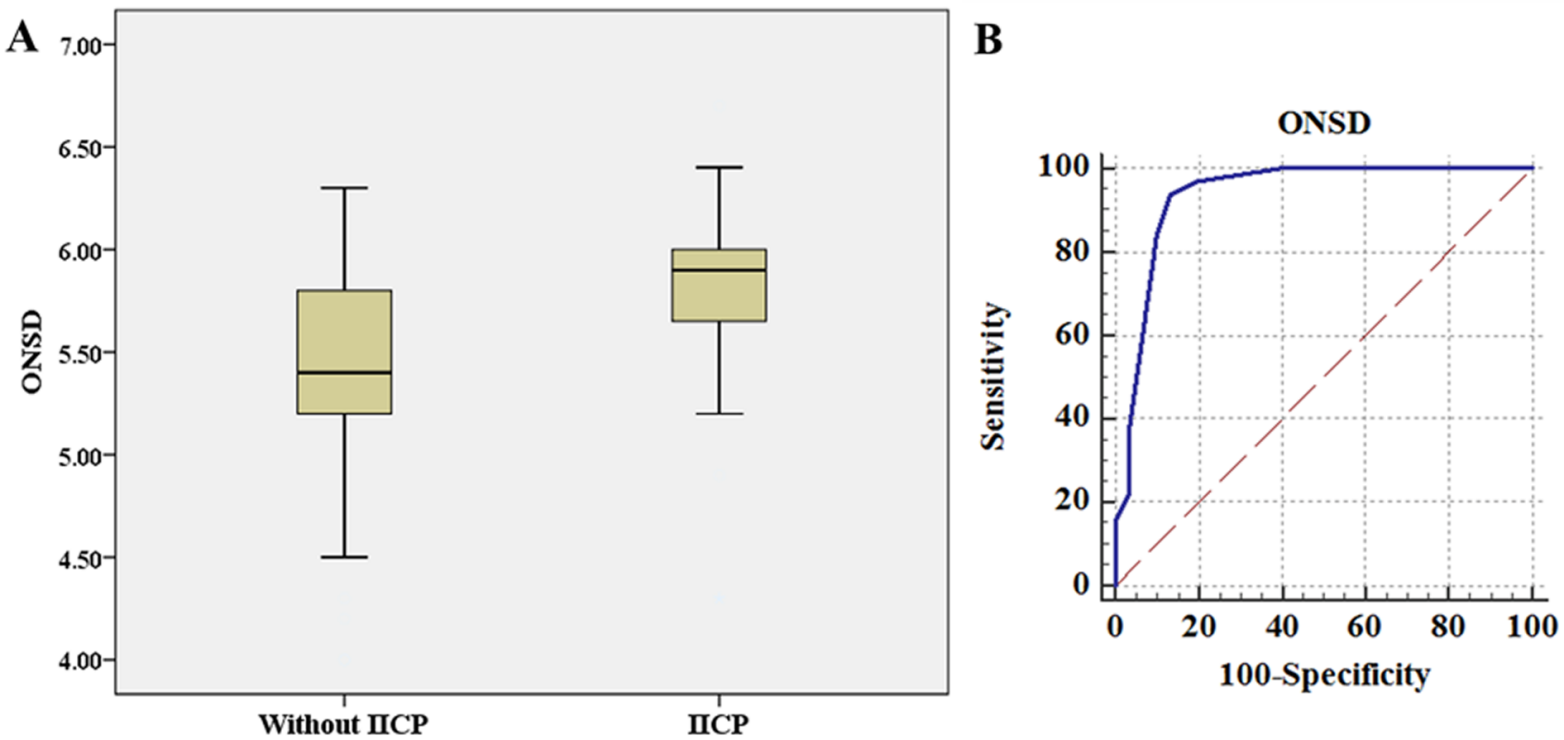
(A): ONSD in groups without IICP and with IICP. The bar represents the median value and the 25th to 75th percentiles. ONSD in patients with IICP (5.80 mm, range 4.3–6.7 mm) is significantly higher than that in those without IICP (5.3 mm, range 4.0–6.2 mm) (*P* < 0.001). (B): The area under the receiver operator characteristic curve is 0.936. ONSD > 5.6 mm yielded a sensitivity of 93.75% (95% CI: 79.2%–99.2%) and a specificity of 86.67% (95% CI: 69.3%–96.2%).

ICC for intra-observer and inter-observer agreement was 0.98 (95% CI: 0.96–0.99) and 0.96 (95% CI: 0.94–0.98), respectively. ICC between right and left ONSD measurments was 0.97 (95% CI: 0.94–0.98). The mean ONSD was 5.80 ± 0.45 mm in the IICP group (> 20 mmHg, 32 measurements), which was significantly higher than that in the low ICP group (mean ONSD = 5.30 ± 0.61 mm, *P* < 0.01) (Fig 1A).

Using ICP as the standard criterion, we created an ROC curve (AUC = 0.936, 95% CI: 0.844–0.983) to establish the optimal cut-off point that would optimize ONSD sensitivity and specificity (Fig 1B). An ONSD of 5.6 mm yielded the most favorable balance of test characteristics, with a sensitivity of 93.75% (95% CI: 79.2%–99.2%) and a specificity of 86.67% (95% CI: 69.3%–96.2%). All relevant data are included in the S1 Table.

**Fig 2:**
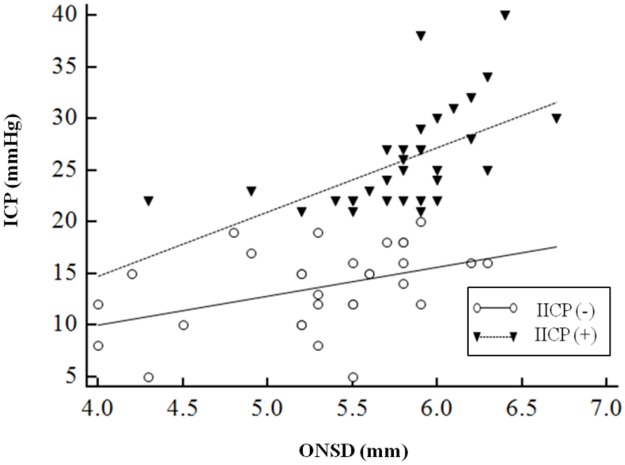
Scatterplot relating optic nerve sheath diameter (ONSD) and intracranial pressure (ICP) according to the presence of increased intracranial pressure (IICP). The linear prediction from regression is shown as solid or dotted line.

Using simple liner regression, there was a linear correlation between ONSD and ICP (r = 0.60, *P* < 0.01). Interestingly, the IICP group showed more significant linear correlation with ONSD (r = 0.57, *P* < 0.01) compared to the non-IICP group (r = 0.42, *P* = 0.02) (Fig 2)

### Illustrative case

**Fig 3:**
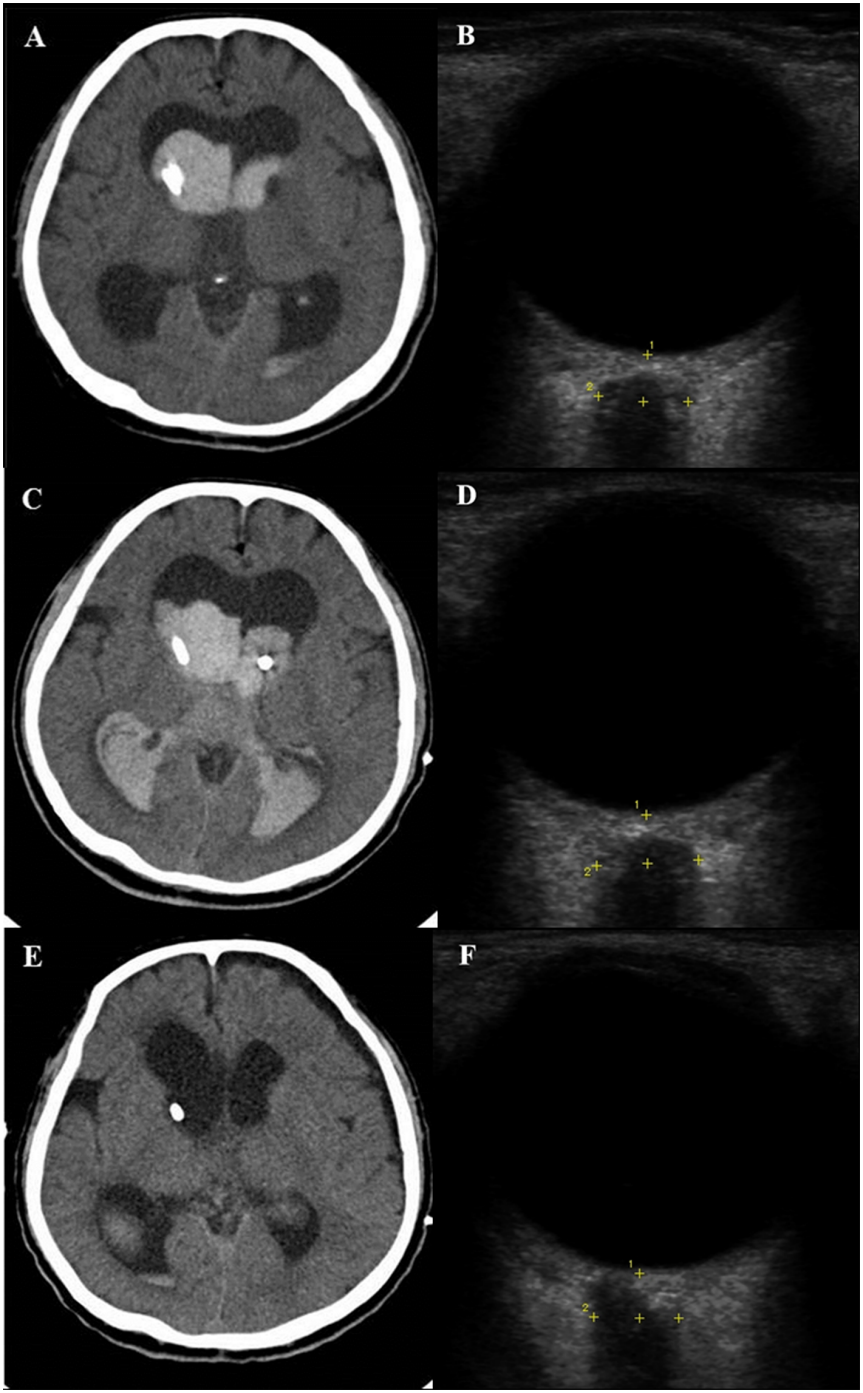
(A, B): A 65-year-old man presented with sudden-onset altered mentality caused by acute IVH and hydrocephalus. US ONSD was measured as 5.7 mm and an EVD was placed in the right lateral ventricle with an opening pressure of 22 mmHg. (C, D): Three hours later, the patient’s ICP surged to 40 mmHg with an ONSD of 6.4 mm due to rebleeding of the right PICA aneurysm. Emergency coil embolization of the right PICA aneurysm was performed and another EVD was inserted in the left lateral ventricle. (E, F): Brain CT scans taken 3 weeks after the operation showed a substantial improvement of hydrocephalus and IVH with an ICP of 10 mmHg and an ONSD of 5.4 mm.

A 65-year-old man presented with a case of sudden-onset altered mentality. Brain CT scans revealed acute IVH from the lateral ventricle to the 4^th^ ventricle with hydrocephalus. US ONSD was 5.7 mm and an EVD was placed in the patient’s right lateral ventricle with an opening pressure of 22 mmHg. Three hours later, the patient’s ICP surged to 40 mmHg with an ONSD of 6.4 mm, and brain CT angiography showed aggravation of IVH due to a ruptured right posterior inferior cerebellar artery (PICA) aneurysm. Emergency coil embolization of the right PICA aneurysm was performed and another EVD was inserted in the left lateral ventricle. The EVD catheter was replaced after 2 weeks and maintained continuously. Brain CT scans taken in the 3rd week after the operation showed substantial improvement of hydrocephalus and IVH with an ICP of 10 mmHg and an ONSD of 5.4 mm (Fig 3).

## Discussion

ONSD as seen on bedside US showed good correlation with directly measured ICP using an EVD catheter (r = 0.77, p < 0.01). The mean diameter of ONSD in patients with increased ICP was 5.80 ± 0.45 mm, which was significantly higher than that in those without increased ICP (5.30 ± 0.61 mm). The optimal cut-off point of ONSD for identifying increased ICP was 5.6 mm, yielding a sensitivity of 93.75% and a specificity of 86.67%.

Non-invasive tests such as transcranial Doppler (TCD), tympanic membrane displacement (TMD), and US have been used to detect IICP. Unlike US, monitoring with TCD and TMD require special equipment and are operator-dependent procedures with a high percentage of unsuccessful measurements, ranging up to 60%. Moreover, TMD testing takes up to 60 min [15–17]. Accordingly, these non-invasive methods are inadequate in urgent situations in the ED or ICU. On the other hand, US measurement of ONSD is reproducible and is an easily learned procedure for physicians with no experience with US, with low intra- and inter-observer variation [18, 19].

Since the optic nerve sheath is continuously connected with the meninges, cerebrospinal fluid (CSF) can move freely between the subarachnoid spaces of the intracranial and intraorbital areas. Therefore, patients who suffer from intracranial hemorrhaging or masses can experience an increase of ONSD due to CSF accumulation [20, 21]. Tayal et al. [5] reported that the mean ONSD associated with increased ICP seen on brain CT was 6.27 mm, which is significantly higher than that seen in patients without abnormal CT findings (mean, 4.94 mm). Blaivas et al. [3] also showed that IICP as demonstrated by CT was associated with an elevated mean ONSD of 6.27 mm (5.6–6.89 mm), in contrast to that in the control group (mean, 4.42 mm). In addition to comparative studies of ONSD and CT findings, direct correlations between ONSD and ICP have been studied. Geeraerts et al. [22] reported that a significant correlation was observed between ONSD and ICP (r = 0.71, p < 0.01). In that study, the optimal cut-off value for identifying increased ICP was 5.86 mm with a sensitivity of 95% and a specificity of 79%. Soldatos et al. found a 0.68 correlation coefficient and an optimal ONSD cut-off point of 5.7 mm in 32 patients with severe traumatic brain injury [23]. According to Amini et al., the correlation coefficient was 0.88 and the cut-off point was 5.5 mm with sensitivity and specificity of 100% [24].

Differences in ethnicity and type of ICP monitor used can limit the usefulness of the previous results in daily practice. Ethnic differences could be a confounding factor in setting an optimal ONSD to define increased ICP in other cohorts. Ballantyne et al. measured 67 adults in the United Kingdom, in whom ONSD ranged from 2.4 to 4.7 mm (mean, 3.2–3.6mm) In a study conducted on 26 Greek adults, ONSD ranged from 2.2 to 4.9 mm (mean, 3.6 mm), which is similar to that found in a previous study in the United Kingdom and a recent study on a Canadian population (mean, 3.68 mm; range, 2.85–4.40 mm) [23, 25]. However, other studies reported higher mean values. Maude et al. reported a mean ONSD of 4.41 mm (range, 4.24–4.83 mm) in healthy volunteers in Bangladesh and another study performed in Iran showed that the mean ONSD was 4.6 mm (range, 3.8–5.4 mm) [24, 26]. In addition, a study in an Italian population revealed a mean ONSD of 5.4 mm and a range of 4.3–7.6 mm [27]. These results suggest that a relatively wide inter-individual range of ONSD may be a result of ethnic diversity and genetic differences. Nevertheless, measuring technique of US could be a contributing factor for relative wide range of ONSD. The mean ONSD seen on MRI or CT differed by 0.2 mm between East Asians and Western population. However, mean ONSD seen on ONSD in East Asia and Western population is about 5.0mm and 3.6mm, respectively (S2 Table). Accordingly, studies focusing on the relationship between measuring technique and ONSD are required further.

Regarding the East Asian population, a recent study conducted on healthy Chinese volunteers reported a mean ONSD of 5.1 mm (range, 4.7–5.4 mm) and a study performed on a Korean population found a mean ONSD of 4.9 mm (range, 4.6–5.2 mm) in the normal control group, similar to that of the healthy Chinese population, and a mean ONSD of 5.9 mm (range, 5.8–6.2 mm) in the IICP group diagnosed using brain CT scans, with a cut-off value of 5.5 mm [7, 8]. However, these studies did not correlate ONSD with directly measured ICP. Though Wang et al. [6] measured ICP directly by lumbar puncture and identified a cut-off value of 4.1 mm for IICP, they did not report a linear correlation of ONSD and ICP, and lumbar puncture tends to overestimate ICP [28, 29]. Our study is the first to demonstrate a correlation between ONSD as measured by US and directly measured ICP in an East Asian population.

The type of ICP monitor used also causes difficulty in interpreting the results of comparative tests of ICP and ONSD. Conventionally, ventricular pressure represents a more global, representative measurement of pressure throughout the intracranial space, since any pressure generated by a focal mass such as a tumor or hemorrhage will be transmitted to the lateral ventricle until equilibrium is reached. On the contrary, because ICP is frequently compartmentalized in cases of focal injury-induced parenchymal pressure, parenchymal ICP measurement is used as a local measurement of a regional phenomenon [10, 30]. ONSD, which is theoretically connected to the subarachnoid space and known as a reflection of global ICP is supposed to have a more significant correlation with ventricular pressure rather than focal parenchymal pressure. Nevertheless, previous authors drew conclusions without considering ICP catheter type and most used parenchymal ICP monitoring alone [19, 23, 31, 32] or in combination with intraventricular monitoring [22, 33, 34], only two studies [35, 36] conducted in the USA have used EVD to measure ICP.

Our results may be more appropriate for patients with a moderate amount of hematoma. In cases of a large amount of hematoma which requires prompt evacuation, an intraventricular ICP probe is placed after decompression; therefore, the opening pressure can be underestimated. Nevertheless, the severity of the brain’s condition in terms of hematoma amount and brain swelling, was not considered when analyzing the direct correlation between ICP and ONSD. To reduce selection bias, we excluded patients with severe IICP which required emergency surgical decompression before ICP monitor insertion. Therefore, an optimal ONSD cut-off point of 5.6 mm may reflect IICP due to moderate hematoma in a Korean population.

US measurement of ONSD is rapid, as it is a bedside procedure that takes approximately 5 minutes to perform [17, 36] and was completed within three minutes in our study. Furthermore, this technique showed a good correlation with ICP and allowed researchers to predict IICP as assessed by CT with a sensitivity of 70–100% and a specificity of 73–95% [3, 5, 37]. Though brain imaging studies, such as CT or MRI, are more accurate means of diagnosing IICP than is US measurement of ONSD, the latter technique can potentially be used as a screening method and for serial monitoring to detect IICP in settings where brain imaging machines are not readily available.

### Limitations

The present study has some limitations. First, only a small population from two centers in Korea were recruited. However, this paper was the first study carried out in an Asian population to determine the direct correlation between simultaneous US measurement of ONSD and direct measurement of ICP. Therefore, a multicenter study in a larger population should be considered to obtain a representative ONSD value for the Asian population. Second, inter-observer variation was a concern because US measurement was performed by two investigators. Bauerle et al. [27] reported that Pearson’s correlation coefficient between two investigators was 0.81 on the right side and 0.84 on the left side, respectively. Ballantyne et al. [18] have found that median inter-observer variation is ± 0.2–0.3 mm. After the first 17 examinations, inter-observer variation has been reduced [18]. Accordingly, we think that the level of inter-observer variation in this study is acceptable.

## Conclusion

ONSD as seen on bedside US correlated well with directly measured ICP in Korean adults with brain lesions. The optimal cut-off point of ONSD for detecting ICP was 5.6 mm. Further larger studies are required to confirm the results.

## Supporting information

S1 Table Data of included patients. (DOCX)

S2 Table Ultrasound measurements of optic nerve sheath diameter (ONSD) of the normal control groups in the previous studies. (DOCX)

## References

[1] Barlow FRCS Philip, Mendelow, Ph.D., F.R.C.S. A. David, Lawrence, M.Sc. Audrey E., Barlow, M.B. Marion, and Rowan, Ph.D., F.Inst.P John O.. Clinical evaluation of two methods of subdural pressure monitoring. Journal of Neurosurgery. 1985;63(4):5. 10.3171/jns.1985.63.4.0578 4032022

[2] Li ZM Q Zhe MD; Zhang Ning MD; Zhao Jun MD; Shen Dongqing MD. Comparison Between Intraventricular and Intraparenchymal Intracranial Pressure Monitoring in Asian Patients With Severe Traumatic Brain Injury. Neurosurgery Quarterly. 2016;26(2):5.

[3] Blaivas M, Theodoro D, Sierzenski PR. Elevated intracranial pressure detected by bedside emergency ultrasonography of the optic nerve sheath. Acad Emerg Med. 2003;10(4):376–81. . 12670853 10.1111/j.1553-2712.2003.tb01352.x

[4] Girisgin AS, Kalkan E, Kocak S, Cander B, Gul M, Semiz M. The role of optic nerve ultrasonography in the diagnosis of elevated intracranial pressure. Emerg Med J. 2007;24(4):251–4. doi: 10.1136/emj.2006.040931 17384377 10.1136/emj.2006.040931 PMC2658229

[5] Tayal VS, Neulander M, Norton HJ, Foster T, Saunders T, Blaivas M. Emergency department sonographic measurement of optic nerve sheath diameter to detect findings of increased intracranial pressure in adult head injury patients. Ann Emerg Med. 2007;49(4):508–14. doi: 10.1016/j.annemergmed.2006.06.040 . 16997419 10.1016/j.annemergmed.2006.06.040

[6] Wang L, Feng L, Yao Y, Wang Y, Chen Y, Feng J, et al Optimal optic nerve sheath diameter threshold for the identification of elevated opening pressure on lumbar puncture in a Chinese population. PLoS One. 2015;10(2):e0117939 doi: 10.1371/journal.pone.0117939 25664663 10.1371/journal.pone.0117939 PMC4322040

[7] Chen H, Ding GS, Zhao YC, Yu RG, Zhou JX. Ultrasound measurement of optic nerve diameter and optic nerve sheath diameter in healthy Chinese adults. BMC Neurol. 2015;15:106 doi: 10.1186/s12883-015-0361-x 26148482 10.1186/s12883-015-0361-x PMC4493801

[8] Lee SU, Jeon JP, Lee H, Han JH, Seo M, Byoun HS, et al Optic nerve sheath diameter threshold by ocular ultrasonography for detection of increased intracranial pressure in Korean adult patients with brain lesions. Medicine (Baltimore). 2016;95(41):e5061 doi: 10.1097/MD.0000000000005061 . 27741121 10.1097/MD.0000000000005061 PMC5072948

[9] Wang L, Feng L, Yao Y, Deng F, Wang Y, Feng J, et al Ultrasonographic Evaluation of Optic Nerve Sheath Diameter among Healthy Chinese Adults. Ultrasound Med Biol. 2016;42(3):683–8. doi: 10.1016/j.ultrasmedbio.2015.11.020 . 26738627 10.1016/j.ultrasmedbio.2015.11.020

[10] Ko S-B. Multimodality Monitoring inthe Neurointensive CareUnit: A Special Perspective for Patients with Stroke. Journal of Stroke. 2013;15(2):10 doi: 10.5853/jos.2013.15.2.99 24324945 10.5853/jos.2013.15.2.99 PMC3779668

[11] Newman WD, Hollman AS, Dutton GN, Carachi R. Measurement of optic nerve sheath diameter by ultrasound: a means of detecting acute raised intracranial pressure in hydrocephalus. Br J Ophthalmol. 2002;86(10):1109–13. 12234888 10.1136/bjo.86.10.1109 PMC1771326

[12] Bauerle J, Schuchardt F, Schroeder L, Egger K, Weigel M, Harloff A. Reproducibility and accuracy of optic nerve sheath diameter assessment using ultrasound compared to magnetic resonance imaging. BMC Neurol. 2013;13:187 doi: 10.1186/1471-2377-13-187 24289136 10.1186/1471-2377-13-187 PMC4219451

[13] Bundgaard H vO G, Larsen KM, Landsfeldt U, Jensen KA, Nielsen E, Cold GE. Effects of sevoflurane on intracranial pressure, cerebral blood flow and cerebral metabolism. A dose-response study in patients subjected to craniotomy for cerebral tumours. Acta Anaesthesiol Scand. 1998;42(6):7. 10.1111/j.1399-6576.1998.tb05292.x 9689265

[14] Cold GE B H, von Oettingen G, Jensen KA, Landsfeldt U, Larsen KM. ICP during anaesthesia with sevoflurane: a dose-response study. Effect of hypocapnia. 1998;71:3. 10.1007/978-3-7091-6475-4_81 9779207

[15] Rosenberg JB S A, Savel RH, Eisen LA. Non-invasive methods of estimating intracranial pressure. Neurocrit Care. 2011;15(3):10. 10.1007/s12028-011-9545-4 21519957

[16] Raboel PH B J Jr, Andresen M, Bellander BM, Romner B. Intracranial Pressure Monitoring: Invasive versus Non-Invasive Methods-A Review. Crit Care Res Pract. 2012;2012:950393 doi: 10.1155/2012/950393 22720148 10.1155/2012/950393 PMC3376474

[17] Kristiansson H N E, Bartek J Jr, Andresen M, Reinstrup P, Romner B. Measuring elevated intracranial pressure through noninvasive methods: a review of the literature. J Neurosurg Anesthesiol. 2013;25(4):14. 10.1097/ANA.0b013e31829795ce 23715045

[18] Ballantyne SA, O'Neill G, Hamilton R, Hollman AS. Observer variation in the sonographic measurement of optic nerve sheath diameter in normal adults. Eur J Ultrasound. 2002;15(3):145–9. . 12423741 10.1016/s0929-8266(02)00036-8

[19] Maissan IM D P, Haitsma IK, Hoeks SE, Gommers D, Stolker RJ. Ultrasonographic measured optic nerve sheath diameter as an accurate and quick monitor for changes in intracranial pressure. Journal of Neurosurgery. 2015;123(3):5. 10.3171/2014.10.JNS141197 25955869

[20] Helmke K, Hansen HC. Fundamentals of transorbital sonographic evaluation of optic nerve sheath expansion under intracranial hypertension II. Patient study. Pediatr Radiol. 1996;26(10):706–10. . 8805600 10.1007/BF01383384

[21] Launey Y, Nesseler N, Le Maguet P, Malledant Y, Seguin P. Effect of osmotherapy on optic nerve sheath diameter in patients with increased intracranial pressure. J Neurotrauma. 2014;31(10):984–8. doi: 10.1089/neu.2012.2829 . 24372319 10.1089/neu.2012.2829

[22] Geeraerts T, Merceron S, Benhamou D, Vigue B, Duranteau J. Non-invasive assessment of intracranial pressure using ocular sonography in neurocritical care patients. Intensive Care Med. 2008;34(11):2062–7. doi: 10.1007/s00134-008-1149-x . 18509619 10.1007/s00134-008-1149-x

[23] Soldatos T, Karakitsos D, Chatzimichail K, Papathanasiou M, Gouliamos A, Karabinis A. Optic nerve sonography in the diagnostic evaluation of adult brain injury. Crit Care. 2008;12(3):R67 doi: 10.1186/cc6897 18477382 10.1186/cc6897 PMC2481450

[24] Amini A, Kariman H, Arhami Dolatabadi A, Hatamabadi HR, Derakhshanfar H, Mansouri B, et al Use of the sonographic diameter of optic nerve sheath to estimate intracranial pressure. Am J Emerg Med. 2013;31(1):236–9. doi: 10.1016/j.ajem.2012.06.025 . 22944553 10.1016/j.ajem.2012.06.025

[25] Goeres P, Zeiler FA, Unger B, Karakitsos D, Gillman LM. Ultrasound assessment of optic nerve sheath diameter in healthy volunteers. J Crit Care. 2016;31(1):168–71. doi: 10.1016/j.jcrc.2015.10.009 . 26596508 10.1016/j.jcrc.2015.10.009

[26] Maude RR, Hossain MA, Hassan MU, Osbourne S, Sayeed KL, Karim MR, et al Transorbital sonographic evaluation of normal optic nerve sheath diameter in healthy volunteers in Bangladesh. PLoS One. 2013;8(12):e81013 doi: 10.1371/journal.pone.0081013 24312515 10.1371/journal.pone.0081013 PMC3846670

[27] Bauerle J, Lochner P, Kaps M, Nedelmann M. Intra- and interobsever reliability of sonographic assessment of the optic nerve sheath diameter in healthy adults. J Neuroimaging. 2012;22(1):42–5. doi: 10.1111/j.1552-6569.2010.00546.x . 21121998 10.1111/j.1552-6569.2010.00546.x

[28] Lenfeldt N K L, Bergenheim AT, Malm J, Eklund A Our study is the first study that demonstrated the correlation between US measurement of ONSD and direct measurement of ICP. Neurology. 2007;68:4.

[29] Warden KF A A, Trobe JD, Hoff JT. Short-term continuous intraparenchymal intracranial pressure monitoring in presumed idiopathic intracranial hypertension. J Neuroophthalmol. 2011;31:4. 10.1097/WNO.0b013e3182183c8d 21483268

[30] Vender J W J, Dhandapani K, McDonnell D. An evaluation and comparison of intraventricular, intraparenchymal, and fluid-coupled techniques for intracranial pressure monitoring in patients with severe traumatic brain injury. J Clin Monit Comput. 2011;25(4):6 doi: 10.1007/s10877-011-9300-6 21938526 10.1007/s10877-011-9300-6

[31] Geeraerts T, Launey Y, Martin L, Pottecher J, Vigue B, Duranteau J, et al Ultrasonography of the optic nerve sheath may be useful for detecting raised intracranial pressure after severe brain injury. Intensive Care Med. 2007;33(10):1704–11. doi: 10.1007/s00134-007-0797-6 . 17668184 10.1007/s00134-007-0797-6

[32] Sekhon MS, Griesdale DE, Robba C, McGlashan N, Needham E, Walland K, et al Optic nerve sheath diameter on computed tomography is correlated with simultaneously measured intracranial pressure in patients with severe traumatic brain injury. Intensive Care Med. 2014;40(9):1267–74. doi: 10.1007/s00134-014-3392-7 . 25034476 10.1007/s00134-014-3392-7

[33] Moretti R, Pizzi B. Optic nerve ultrasound for detection of intracranial hypertension in intracranial hemorrhage patients: confirmation of previous findings in a different patient population. J Neurosurg Anesthesiol. 2009;21(1):16–20. . 19098619 10.1097/ANA.0b013e318185996a

[34] Moretti R, Pizzi B, Cassini F, Vivaldi N. Reliability of optic nerve ultrasound for the evaluation of patients with spontaneous intracranial hemorrhage. Neurocrit Care. 2009;11(3):406–10. doi: 10.1007/s12028-009-9250-8 . 19636971 10.1007/s12028-009-9250-8

[35] Frumin E, Schlang J, Wiechmann W, Hata S, Rosen S, Anderson C, et al Prospective analysis of single operator sonographic optic nerve sheath diameter measurement for diagnosis of elevated intracranial pressure. West J Emerg Med. 2014;15(2):217–20. doi: 10.5811/westjem.2013.9.16191 24672615 10.5811/westjem.2013.9.16191 PMC3966440

[36] Kimberly HH, Shah S, Marill K, Noble V. Correlation of optic nerve sheath diameter with direct measurement of intracranial pressure. Acad Emerg Med. 2008;15(2):201–4. doi: 10.1111/j.1553-2712.2007.00031.x . 18275454 10.1111/j.1553-2712.2007.00031.x

[37] Komut E, Kozaci N, Sonmez BM, Yilmaz F, Komut S, Yildirim ZN, et al Bedside sonographic measurement of optic nerve sheath diameter as a predictor of intracranial pressure in ED. Am J Emerg Med. 2016;34(6):963–7. doi: 10.1016/j.ajem.2016.02.012 . 26944107 10.1016/j.ajem.2016.02.012

