# Supplementary material for: Correlation of optic nerve sheath diameter with directly measured intracranial pressure in Korean adults using bedside ultrasonography
Source: PLoS One. 2017 Sep 13;12(9):e0183170. doi: 10.1371/journal.pone.0183170 (PMC5597178; doi:10.1371/journal.pone.0183170)
Supplement: S1 Table — (DOCX) [file pone.0183170.s001.docx]

| **S1 Table. Data of included patients** | | | | | | | | |
| --- | --- | --- | --- | --- | --- | --- | --- | --- |
| **Patient No** | **Sex** | **Age** | **ICP (mmHg)** | **ONSD (mm)** | | | | |
|  |  |  |  | **1^st^ Measurement** | | **2^nd^ Measurement** | | **Mean** |
|  |  |  |  | **Right** | **Left** | **Right** | **Left** |  |
| 1 | F | 67 | 15 | 5.2 | 5.2 | 5.2 | 5.2 | 5.2 |
| 2 | F | 52 | 22 | 5.8 | 5.7 | 6.0 | 5.7 | 5.8 |
| 3 | F | 56 | 30 | 6.0 | 6.0 | 6.0 | 6.0 | 6.0 |
| 4 | F | 52 | 25 | 6.3 | 6.3 | 6.3 | 6.3 | 6.3 |
| 5 | M | 51 | 10 | 4.6 | 4.5 | 4.5 | 4.4 | 4.5 |
| 6 | F | 45 | 22 | 6.1 | 5.9 | 6.0 | 6.0 | 6.0 |
| 7 | M | 66 | 18 | 5.8 | 5.8 | 5.8 | 5.8 | 5.8 |
| 8 | M | 65 | 22 | 5.7 | 5.7 | 5.7 | 5.7 | 5.7 |
| 9 | F | 47 | 23 | 5.6 | 5.6 | 5.6 | 5.6 | 5.6 |
| 10 | F | 49 | 34 | 6.3 | 6.3 | 6.3 | 6.3 | 6.3 |
| 11 | M | 49 | 12 | 5.4 | 5.3 | 5.3 | 5.2 | 5.3 |
| 12 | M | 70 | 40 | 6.4 | 6.4 | 6.4 | 6.4 | 6.4 |
| 13 | F | 49 | 12 | 5.5 | 5.5 | 5.5 | 5.5 | 5.5 |
| 14 | F | 68 | 24 | 5.9 | 6.2 | 5.8 | 6.1 | 6.0 |
| 15 | F | 63 | 31 | 6.1 | 6.1 | 6.1 | 6.1 | 6.1 |
| 16 | F | 61 | 24 | 5.6 | 5.7 | 5.8 | 5.7 | 5.7 |
| 17 | F | 83 | 8 | 3.9 | 4.1 | 3.9 | 4.1 | 4.0 |
| 18 | F | 60 | 22 | 5.9 | 5.9 | 5.9 | 5.9 | 5.9 |
| 19 | F | 53 | 15 | 5.6 | 5.6 | 5.6 | 5.6 | 5.6 |
| 20 | F | 72 | 32 | 6.4 | 6.0 | 6.3 | 6.1 | 6.2 |
| 21 | F | 47 | 8 | 5.2 | 5.3 | 5.3 | 5.4 | 5.3 |
| 22 | M | 59 | 18 | 5.7 | 5.7 | 5.7 | 5.7 | 5.7 |
| 23 | M | 44 | 15 | 5.4 | 5.7 | 5.6 | 5.7 | 5.6 |
| 24 | F | 67 | 15 | 4.3 | 4.1 | 4.2 | 4.2 | 4.2 |
| 25 | F | 63 | 17 | 5.0 | 4.8 | 5.0 | 4.8 | 4.9 |
| 26 | F | 29 | 26 | 5.8 | 5.8 | 5.8 | 5.8 | 5.8 |
| 27 | M | 61 | 19 | 4.8 | 4.8 | 4.8 | 4.8 | 4.8 |
| 28 | M | 73 | 21 | 5.9 | 6.0 | 5.8 | 5.9 | 5.9 |
| 29 | F | 22 | 25 | 6.1 | 5.9 | 6.1 | 5.9 | 6.0 |
| 30 | F | 62 | 10 | 5.2 | 5.2 | 5.2 | 5.2 | 5.2 |
| 31 | F | 66 | 10 | 5.4 | 5.1 | 5.3 | 5.0 | 5.2 |
| 32 | F | 58 | 15 | 5.2 | 5.2 | 5.2 | 5.2 | 5.2 |
| 33 | F | 37 | 22 | 5.4 | 5.4 | 5.4 | 5.4 | 5.4 |
| 34 | F | 64 | 25 | 5.8 | 5.8 | 5.8 | 5.8 | 5.8 |
| 35 | M | 41 | 38 | 5.9 | 5.9 | 5.9 | 5.9 | 5.9 |
| 36 | M | 21 | 30 | 6.7 | 6.8 | 6.6 | 6.7 | 6.7 |
| 37 | F | 74 | 16 | 6.4 | 6.3 | 6.2 | 6.3 | 6.3 |
| 38 | F | 50 | 28 | 6.2 | 6.2 | 6.2 | 6.2 | 6.2 |
| 39 | M | 58 | 27 | 5.7 | 5.7 | 5.7 | 5.7 | 5.7 |
| 40 | F | 61 | 27 | 5.8 | 5.9 | 5.7 | 5.8 | 5.8 |
| 41 | M | 80 | 29 | 6.0 | 5.7 | 6.0 | 5.9 | 5.9 |
| 42 | F | 75 | 27 | 6.0 | 5.8 | 6.0 | 5.8 | 5.9 |
| 43 | F | 58 | 12 | 5.9 | 6 | 5.7 | 6 | 5.9 |
| 44 | M | 67 | 13 | 5.3 | 5.3 | 5.3 | 5.3 | 5.3 |
| 45 | M | 57 | 5 | 4.3 | 4.3 | 4.3 | 4.3 | 4.3 |
| 46 | F | 67 | 23 | 5.0 | 4.9 | 4.9 | 4.8 | 4.9 |
| 47 | M | 55 | 14 | 5.6 | 6.0 | 5.7 | 5.9 | 5.8 |
| 48 | F | 59 | 16 | 5.5 | 5.6 | 5.4 | 5.5 | 5.5 |
| 49 | F | 45 | 22 | 5.7 | 5.4 | 5.6 | 5.2 | 5.5 |
| 50 | F | 71 | 16 | 6.2 | 6.2 | 6.2 | 6.2 | 6.2 |
| 51 | M | 51 | 12 | 5.5 | 5.5 | 5.5 | 5.5 | 5.5 |
| 52 | F | 23 | 22 | 4.3 | 4.3 | 4.3 | 4.3 | 4.3 |
| 53 | M | 27 | 16 | 5.8 | 5.8 | 5.8 | 5.8 | 5.8 |
| 54 | F | 66 | 5 | 5.5 | 5.5 | 5.5 | 5.5 | 5.5 |
| 55 | F | 32 | 12 | 3.8 | 4.2 | 3.8 | 4.2 | 4.0 |
| 56 | F | 54 | 22 | 5.4 | 5.6 | 5.3 | 5.7 | 5.5 |
| 57 | F | 74 | 27 | 5.9 | 5.9 | 5.9 | 5.9 | 5.9 |
| 58 | F | 74 | 18 | 5.8 | 5.8 | 5.8 | 5.8 | 5.8 |
| 59 | F | 36 | 19 | 5.1 | 5.5 | 5.1 | 5.5 | 5.3 |
| 60 | M | 50 | 20 | 5.9 | 5.9 | 5.9 | 5.9 | 5.9 |
| 61 | M | 56 | 21 | 5.2 | 5.2 | 5.2 | 5.2 | 5.2 |
| 62 | F | 50 | 21 | 5.5 | 5.5 | 5.5 | 5.5 | 5.5 |
| No, number; F, female; M, male; ICP, intracranial pressure; ONSD, optic nerve sheath diameter | | | | | | | | |
