# Supplementary material for: Correlation of optic nerve sheath diameter with directly measured intracranial pressure in Korean adults using bedside ultrasonography
Source: PLoS One. 2017 Sep 13;12(9):e0183170. doi: 10.1371/journal.pone.0183170 (PMC5597178; doi:10.1371/journal.pone.0183170)
Supplement: S2 Table — (DOCX) [file pone.0183170.s002.docx]

| **S2 Table. Ultrasound measurements of optic nerve sheath diameter (ONSD) of the normal control groups in the previous studies** | | | | | |
| --- | --- | --- | --- | --- | --- |
| **Author (year)** | **Country** | **No** | **Mean (range)**  **(mm)** | **Probe**  **(MHz)** | **Control group** |
| **Western** |  |  |  |  |  |
| Ballantyne (2002)［1］ | UK | 67 | 3.2 - 3.6 (2.4-4.7) | 7 | Normal adults |
| Soldatos (2008)［2］ | GRC | 26 | 3.6 (2.2-4.9) | 9 | Pts. without brain injury |
| Goeres (2016)［3］ | CAN | 120 | 3.68 (2.85-4.4) | 13-16 | Healthy volunteer |
| **West Asia** |  |  |  |  |  |
| Amini (2013)［4］ | IRN | 36 | 4.6 (3.8-5.4) | 7.5 | Pts. with normal LP pressure |
| **South Asia** |  |  |  |  |  |
| Maude (2013)［5］ | BGD | 136 | 4.41 (4.25-4.75) | 15 | Healthy volunteer |
| **East Asia** |  |  |  |  |  |
| Chen (2015)［6］ | CHN | 519 | 5.1 (4.7-5.4) | 12-13 | Healthy volunteer |
| Lee (2016)［7］ | KOR | 134 | 4.9 (4.6-5.2) | 13 | Pts. with back pain or health check-up participants |
| No, number; UK, United Kingdom; BGD, Bangladesh; GRC, Greece; CAN, Canada; IRN, Iran; KOR, Korea; CHN, China; pts, patients; LP, lumbar puncture | | | | | |

**Reference**

1. Ballantyne SA, O'Neill G, Hamilton R, Hollman AS. Observer variation in the sonographic measurement of optic nerve sheath diameter in normal adults. Eur J Ultrasound. 2002;15(3):145-9.

2. Soldatos T, Karakitsos D, Chatzimichail K, Papathanasiou M, Gouliamos A, Karabinis A. Optic nerve sonography in the diagnostic evaluation of adult brain injury. Crit Care. 2008;12(3):R67.

3. Goeres P, Zeiler FA, Unger B, Karakitsos D, Gillman LM. Ultrasound assessment of optic nerve sheath diameter in healthy volunteers. J Crit Care. 2016;31(1):168-71.

4. Amini A, Kariman H, Arhami Dolatabadi A, Hatamabadi HR, Derakhshanfar H, Mansouri B, et al. Use of the sonographic diameter of optic nerve sheath to estimate intracranial pressure. Am J Emerg Med. 2013;31(1):236-9.

5. Maude RR, Hossain MA, Hassan MU, Osbourne S, Sayeed KL, Karim MR, et al. Transorbital sonographic evaluation of normal optic nerve sheath diameter in healthy volunteers in Bangladesh. PLoS One. 2013;8(12):e81013.

6. Chen H, Ding GS, Zhao YC, Yu RG, Zhou JX. Ultrasound measurement of optic nerve diameter and optic nerve sheath diameter in healthy Chinese adults. BMC Neurol. 2015;15:106.

7. Lee SU, Jeon JP, Lee H, Han JH, Seo M, Byoun HS, et al. Optic nerve sheath diameter threshold by ocular ultrasonography for detection of increased intracranial pressure in Korean adult patients with brain lesions. Medicine (Baltimore). 2016;95(41):e5061.
